# What is next for Hematology, Hemotherapy and Cell Therapy? A message and an invitation from incoming editors

**DOI:** 10.1016/j.htct.2026.106267

**Published:** 2026-02-05

**Authors:** Erich V. De Paula, Carla Luana Dinardo

**Affiliations:** a Faculdade de Ciências Médicas, Universidade Estadual de Campinas (FCM - Unicamp), Campinas, SP, Brazil; b Fundação Pró-sangue Hemocentro de São Paulo (Pró-sangue), São Paulo, SP, Brazil

Along with the start of 2026, we assume the great responsibility of continuing the work of editors who in the past brought our journal *Hematology, Hemotherapy and Cell Therapy* (HTCT) to the place it occupies today in the scientific publishing landscape. Throughout this time, the journal has achieved national and international recognition, illustrated, but not exclusively defined, by indexing and impact metrics. It is fair to say today that, nationally, HTCT has ceased to be a channel for publishing articles of local interest, becoming a privileged destination for disseminating national scientific publications. And internationally, the journal's visibility is confirmed by the year-on-year increase in the number of submissions, which reached 646 in 2025, compared to 412 in 2021. The quality of peer review has followed this trend, leading to an acceptance rate of 17% of submitted articles in the last year, further illustrating the challenge faced by our editorial board. Finally, in addition to this list of HTCT's qualities, we have what is perhaps our greatest asset: the exclusively scientific basis of its editorial policy, in an environment where other interests compete with science in the publishing industry. In this scenario of growth and consolidation, it is important that, as we assume this responsibility, we ask ourselves, and bring to the scientific community linked to HTCT the question: what new paths should be followed?

Having posed the question, we take the liberty of presenting some of the impressions that guide us in this endeavor. The first is to maintain and expand the relevance of HTCT in the global environment scientific publications in our field, which is broadly speaking, a scenario that has been undergoing important changes. According to a report prepared by Carlos Henrique Brito Cruz for the UN Multistakeholder Forum on Science, Technology and Innovation (2025), in the year 2024, 60% of scientific articles published worldwide had authors from low- and middle-income countries, a stark contrast to what occurred 30 years ago, when 87% of publications originated from wealthy countries.[1] This change is not only demographic, but also brings with it a broadening of scientific agendas, represented by the yet timid incorporation of themes such as the management of global health systems, innovation in resource-limited contexts, “One Health”, and neglected diseases. Cross-cutting themes such as education in healthcare profession, disease burden in under-represented epidemiological scenarios, mental health and well-being consequences of disease, and solutions for incorporating new technologies such as AI are also gaining strength in this process, without prejudice to the fundamental themes of science in our area such as basic science, translational, epidemiology, and clinical intervention studies. In this scenario, we believe that increasing the visibility and relevance of HTCT involves attracting and incorporating high-quality publications that bring together these emerging areas with our core essential interest, Hematology, Hemotherapy, and Cell Therapy, which will always be protected and guide our editorial line. In fact, new scientific journals from highly relevant entities in our field have been recently vocalizing similar objectives.

At the same time, we understand that the link of HTCT to scientific entities of recognized seriousness and the possibility of subsidized open access is an element that should be further explored, as a guarantee of HTCT's scientific seriousness in attracting publications from the most affluent and traditional countries in science in our area, as well as in consolidating HTCT as the preferred publication vehicle for high-quality articles produced in Brazil and in the countries most directly linked to our community, whose quality of scientific production in hematology is growing more and more.[2] Here, it is also worth highlighting the recent changes in the Brazilian national policy for evaluating postgraduate programs, which modulates the emphasis on scientific journal impact metrics, beginning to value other elements that, in our view, are already being progressively more recognized in HTCT.

Finally, considering the increasingly important role of social networks as an environment for discussion and dissemination of scientific production, it is part of our objectives to seek strategies to further integrate HTCT into this environment.

For all these objectives, we need the collaboration of the entire community currently connected to HTCT. The generous contribution of those who are invited to act as reviewers. The tireless commitment of our editorial board, in the pursuit of both quality and speed in the evaluation of submissions, taking as a reference the deference of the position attributed to them by our entire community. And to everyone, in the invitation to consider HTCT more and more as a vehicle for publishing their best articles and reviews.

We conclude by inviting you to express your suggestions and criticisms through our contacts. And reiterating our most sincere commitment to contributing to this new phase of HTCT.

## Data availability statement

N/A.

## Conflicts of interest

The authors declare no conflicts of interest.
